# On the control of secondary carbanion structure utilising ligand effects during directed metallation

**DOI:** 10.3762/bjoc.8.5

**Published:** 2012-01-09

**Authors:** Andrew E H Wheatley, Jonathan Clayden, Ian H Hillier, Alison Campbell Smith, Mark A Vincent, Laurence J Taylor, Joanna Haywood

**Affiliations:** 1 Department of Chemistry, University of Cambridge, Lensfield Road, Cambridge, CB2 1EW, UK; 2 School of Chemistry, University of Manchester, Oxford Road, Manchester, M13 9PL, UK

**Keywords:** directed metallation, Lewis base, ligand effects, lithium, secondary carbanion

## Abstract

*N*,*N*-Diisopropyl-2-propylbenzamide **6**-H undergoes lateral deprotonation by *t-*BuLi in the presence of the Lewis base PMDTA (*N*,*N*,*N′*,*N″*,*N″*-pentamethyldiethylenetriamine) to give a benzyllithium **6**-Li*_l_*·PMDTA that incorporates a trigonal planar secondary carbanion. In the solid state, the amide directing group and the PMDTA additive work together to abstract the metal ion from the deprotonated α-C of the propyl group (4.107(4) Å). A short distance of 1.376(3) Å is observed between the deprotonated carbon centre and a planar aromatic system that shows a pattern of bond lengths which contrasts with that reported for related tertiary carbanion systems. Analogous benzylic deprotonation is seen if **6**-H is treated with *t*-BuLi in the presence of diglyme to give **6**-Li*_l_*·DGME. X-ray crystallography now shows that the metal ion more closely approaches the tertiary carbanion (2.418(6) Å) but that the planarity of the deprotonated carbon centre and the bonding pattern in the organic anion seen in the PMDTA complex are retained. DFT analysis corroborates both the short distance between aromatic ring and carbanion centre and the unperturbed nature of aromaticity in **6**-Li*_l_*·L (L = Lewis base). The observation of two structure-types for the carbanion in solution is explained theoretically and by NMR spectroscopy in terms of *cis* and *trans* isomerism imparted by partial double bond character in the arene–(α-C) bond.

## Introduction

Directed deprotonative lithiation – where the directing group generally combines inductive electron withdrawal with the presence of an electron-rich metal-coordinating atom [1] – has established itself as an enormously powerful tool for the elaboration of aromatic and heteroaromatic compounds [2–9] and continues to find new applications today [10–11]. Various highly selective aryl C–H deprotonative metallations have been reported because of the directing group's ability to inductively raise hydrogen atom acidity and because the incoming organometallic reagent closely approaches the reactive position (“*ortho* lithiation” when deprotonation occurs adjacent to the directing group [2–3], “directed remote metallation” when reaction is non-adjacent [12–15]).

However, the presence of substituents at the *ortho* position of the aromatic ring introduces the possibility of deprotonating the substituent at the benzylic (or α-) position (“lateral lithiation”) through the directing group coordinating the incoming organometallic substrate whilst conjugatively withdrawing electrons from the benzylic group [2]. In the case of lateral reaction, stabilisation of the resulting anion is expected to favour benzylic deprotonation when reactions are potentially competitive [16–17]. However, in practice, the exact nature of the lateral group may complicate matters. For example, the presence of a heteroatom at the β-position of the lateral group may either promote or retard lateral deprotonation on electronic grounds [1–2]. Moreover, steric effects have also been found to play an important role. Hence, whereas the formation of primary [18] and secondary [19] carbanions through lateral deprotonation has been known for many years, it is only very recently that the analogous formation of tertiary carbanions has been reported [20].

**Scheme 1:**
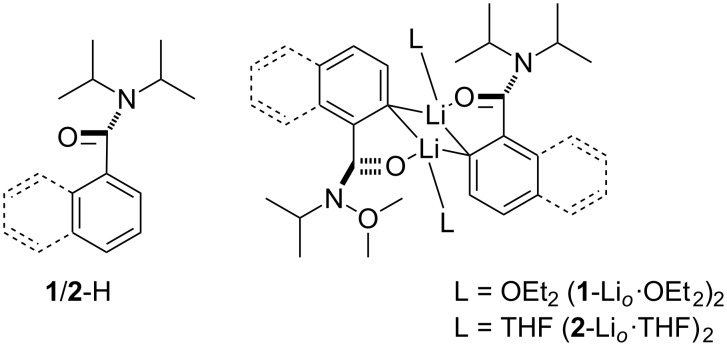
Molecular structures of **1**/**2**-H and their corresponding *ortho*-lithiates [21].

Amongst the most important, versatile and widely used agents for directing the deprotonative lithiation of aromatic substrates are amide type (or “N+O”) groups [3]. In the case of such systems, solid-state structural evidence for carbanion structure has only emerged in the last decade or so. These data focused on reactions directed by tertiary amides; with the *ortho* metallation of *N*,*N*-diisopropylbenzamide **1**-H and its naphthamide analogue **2**-H [21] giving species that have been characterised as solid-state dimers. These metallo-intermediates are each based on a (CLi)_2_ core wherein each metal ion is further stabilised by one solvent molecule (OEt_2_ or THF, Scheme 1) and an amide O-centre through modulation of the amide–arene twist angle (documented as being near perpendicular in the corresponding arylamide substrates [22]).

**Scheme 2:**
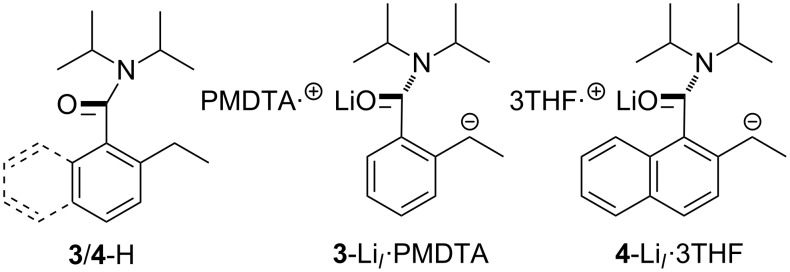
Molecular structures of **3**/**4**-H and their corresponding lateral lithiates [23–24].

**Scheme 3:**
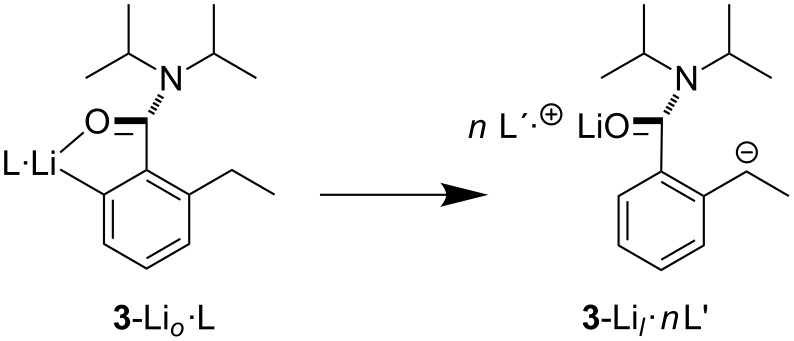
Conversion of kinetic *ortho*-lithiate into the thermodynamic lateral lithiate under the influence of strong (L = THF, *n* = 1, L′ = PMDTA) or excess (e.g., L = L′ = THF, *n* > 1) Lewis base [23].

More recently, the focus has shifted towards the study of the competition between the *ortho* and lateral deprotonation of 2-alkylated benzamide substrates. Hence, 2-ethylated analogues of **1**-H and **2**-H (**3**-H and **4**-H, respectively; Scheme 2) have been treated with *t*-BuLi in the presence of either PMDTA (*N*,*N*,*N′*,*N″*,*N″*-pentamethyldiethylenetriamine) (for **3**-H) [23] or THF (for **4**-H) [24] to yield **3**/**4**-Li*_l_*·*n*L (*n* = 1, L = PMDTA; *n* = 3, L = THF). Notably, whereas the deptonation of **4**-H in the presence of THF yielded the lateral lithiate as a tris(THF) solvate, the tridentate electron donor PMDTA was required to replicate this chemistry with **3**-H. Attempts to utilise THF in this last system resulted, instead, in the isolation and characterisation of *ortho* metallated **3**-Li*_o_*·THF, the solid-state structure of which revealed a dimeric structure analogous to those seen for **1**-Li*_o_*·THF and **2**-Li*_o_*·THF [21]. It was subsequently established that the conversion of this (kinetic) lithiate into the thermodynamic (lateral) species could be observed in solution spectroscopically (Scheme 3) [23].

**Scheme 4:**
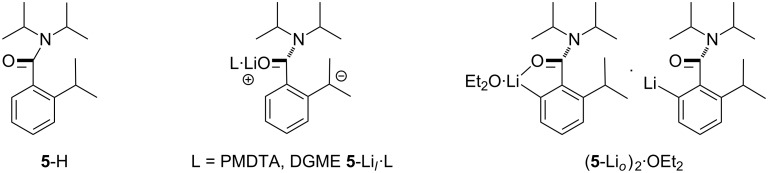
Molecular structure of **5**-H and its lateral and *ortho*-lithiates [20].

The need to utilise multidentate donors in place of monodentate solvents such as OEt_2_ or THF to incur the lateral deprotonation of 2-alkylated arylamides in which the 6-position is not blocked (contrast this scenario with the conversion of **4**-H into **4**-Li*_l_*·3THF) recently led us to examine the hitherto unachievable formation of tertiary carbanions by directed lateral lithiation [16–17]. Accordingly, 2-isopropyl-*N*,*N*-diisopropylbenzamide (**5**-H) has been used to source **5**-Li*_l_*·L (L = PMDTA, DGME; DGME = diglyme) and also, under kinetic control, the remarkable hemi-solvated *ortho*-lithiate (Scheme 4). This development allowed thermodynamic lithiate **5**-Li*_l_*·PMDTA to be successfully used to generate a variety of benzamides bearing quaternary C-centres at the aromatic 2-position [20].

**Scheme 5:**
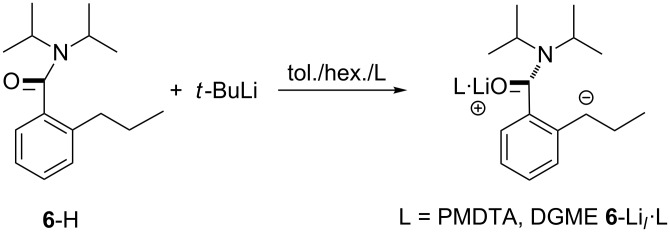
Lateral metallation of **6**-H using *t*-BuLi in the presence of Lewis base L.

In this work, we revisit the formation of secondary carbanions, reporting variously solvated lateral lithiates of *N*,*N*-diisopropyl-2-propylbenzamide **6**-H. We had previously prepared this compound as part of a study of the conformational stability and lability of tertiary aromatic amides bearing a single *ortho* substituent [25]. At ambient temperature, amides with this substitution pattern are conformationally mobile about their aryl–CO bond [26] on a timescale of seconds or less, and hence, inseparable into atropisomers [27]. However, at the temperatures commonly used to effect lithiation reactions, they should exist as chiral, racemic atropisomers. The stepwise lateral ethylation/*ortho* methylation of **3**-H and lateral methylation/*ortho* methylation of **6**-H [17] prior to warming returned conformationally rigid but oppositely configured amides with diastereomeric ratios >9:1; establishing that reaction was kinetically controlled [28]. Present structural studies of lateral lithiates of **6**-H reveal extensive charge delocalisation from a trigonal planar carbanion centre; the displacement of the metal from which is strongly solvent dependent (Scheme 5). Moreover, the improved quality of the crystallographic data for **6**-Li*_l_*·L relative to that achievable for **3**-Li*_l_*·PMDTA reveals significant structural parameters that are not available from the previously characterised secondary carbanion [23].

## Results and Discussion

### Solid-state structural studies

**Figure 1:**
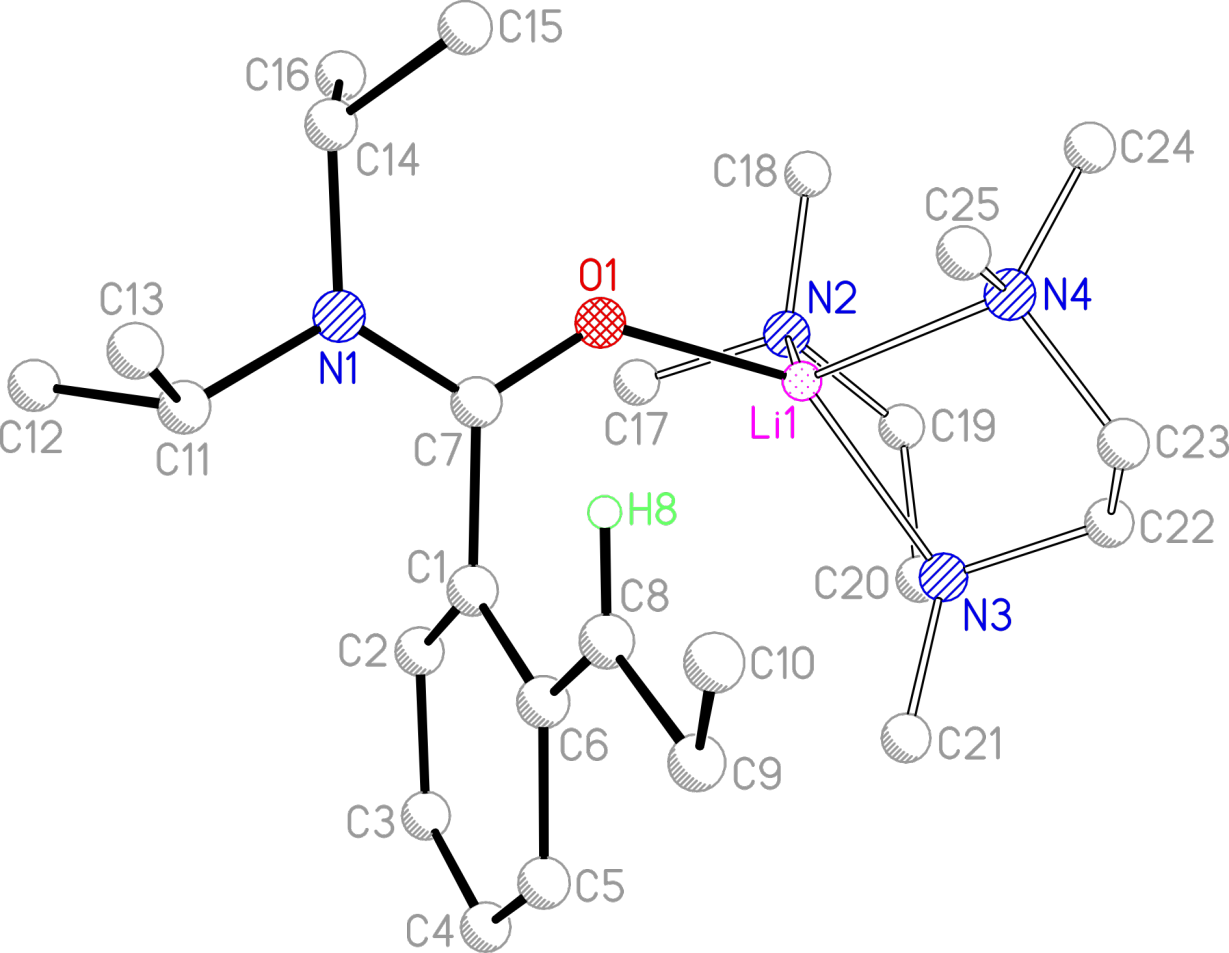
Molecular structure of **6**-Li*_l_*·PMDTA; H-atoms (excl. H8) omitted for clarity. Selected bond lengths (Å) and angles (°): O1–Li1 1.845(3), N2–Li1 2.116(4), N3–Li1 2.097(3), N4–Li1 2.123(4), C8^…^Li1 4.107(4), C8–H8 0.957(19), C8–C9 1.497(3), C9–C10 1.510(3), C8–C6 1.376(3), C6–C5 1.450(3), C5–C4 1.371(3), C4–C3 1.371(3), C3–C2 1.405(3), C2–C1 1.372(3), C1–C6 1.446(3), C6–C1–C7–O1 88.4(2), C7–C1–C6–C8 6.1(3).

Based on our knowledge of the ability of Lewis base solvent or additives to influence the chemoselectivity of lithiation, **6**-H was treated at −78 °C with *t-*BuLi in a hydrocarbon medium to which 1 equiv of PMDTA had been added. The resulting dark purple solution was transferred directly to a freezer (−30 °C) whereupon storage afforded a product that X-ray crystallography revealed to be benzylically deprotonated **6**-Li*_l_*·PMDTA; in which the metal is encapsulated by the amide oxygen atom and the three donor sites of PMDTA (Scheme 5, Figure 1 and Supporting Information File 1). Importantly, the use of a Pr substituent (cf. 2-Et in **3**-H) obviates the crystallographic disorder that limited the analysis of the structure of **3**-Li*_l_*·PMDTA by preventing the anisotropic refinement of both the aromatic ring and the deprotonated alkyl chain [23]. In fact, the direct observation of H8 by Fourier difference synthesis [29–30] and the anisotropic refinement of this atom allows an exact understanding of the geometry at the carbanion centre: In **6**-Li*_l_*·PMDTA amide coordination and solvation by PMDTA have worked together to displace the Li^+^ ion from deprotonated C8, resulting in an essentially flat secondary carbanion (C6–C8–C9 125.0(2)°, C6–C8–H8 120.9(11)°, C9–C8–H8 114.0(11)°).

**Scheme 6:**
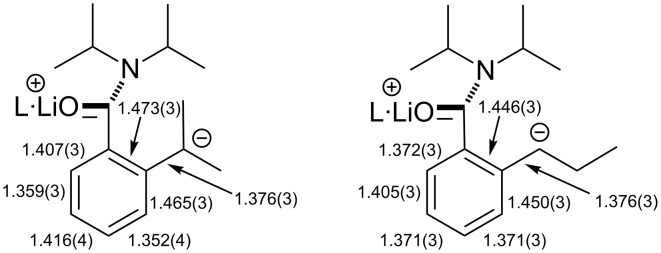
Comparison of aromatic and aryl–(α-C) bond distances in **5**-Li*_l_*·L [20] and **6**-Li*_l_*·L (L = PMDTA).

Akin to the recently reported structure of **5**-Li*_l_*·PMDTA, a short arene–(α-C) bond is noted in **6**-Li*_l_*·PMDTA (1.376(3) Å). However, whereas the amide–arene orientation in a laterally lithiated tertiary aromatic amide has previously approximated to 80° in the presence of a secondary benzylic carbanion (viz. C(CLi)–C–C–O 79.2(4)° in **3**-Li*_l_*·PMDTA [23], 82.5(5)° in **4**-Li*_l_*·3THF [24]) and has been significantly reduced (to 42.8(3) and 39.7(2) in **5**-Li*_l_*·L (L = PMDTA, DGME) [20]) in the presence of a tertiary benzylic carbanion, that in **6**-Li*_l_*·PMDTA is higher (C6–C1–C7–O1 88.4(2)°), plainly precluding any azaenolate contribution to anion stability (C7–C1 1.493(3) Å). Moreover, though the analysis of charge delocalisation has recently been undertaken for benzylic tertiary carbanions, revealing a pentadienyl bonding pattern with alternating short and long bonds noted between the deprotonated (α-C) and the aromatic carbon centre *para* to it, the situation for secondary carbanionic **6**-Li*_l_*·PMDTA is different. This could not be ascertained for previously reported **3**-Li*_l_*·PMDTA [23], where crystallographic disorder meant that both the arene–(α-C) interaction and bonds within the aromatic ring were constrained. In the present case, both aromatic and lateral group carbon atoms could be refined freely, allowing us to compare bond lengths in *tert*-carbanion **5**-Li*_l_*·PMDTA and *sec*-carbanion **6**-Li*_l_*·PMDTA in Scheme 6. It is clear that bond–length alternation seen in **5**-Li*_l_*·PMDTA (where the carbanion is best viewed as exhibiting pentadienyl character) is not replicated in **6**-Li*_l_*·PMDTA. Instead, combined with the planarity of the ring system observed here, bond lengths in **6**-Li*_l_*·PMDTA suggest the retention of aromaticity. Consistent with this, a torsion of only 6.1(3)° between directing and deprotonated groups (C(=O)–C–C–C(Li)) in **6**-Li*_l_*·PMDTA contrasts with one of 32.6° in **5**-Li*_l_*·PMDTA. The behaviour of the present system therefore contrasts with that reported previously for benzylically deprotonated analogues. Thus, benzyl anions [31–43] including amino- [44–46], phosphino- [47–49], thio-/sulfamido-/sulfimido- [50–53] and silylbenzyl anions [54–62], have all demonstrated long aryl–(α-C) distances (1.419–1.538 Å) with retention of aromaticity. In contrast, **5**-Li*_l_*·PMDTA and laterally lithiated *N*-trimethylsilyl *o*-methylphenyldiphenylphosphinimine [63] demonstrated a short aryl-(α-C) distance (of 1.376(6) Å in both cases [20,63]) in tandem with a pentadienyl bonding pattern and significant perturbation of the aryl system. Uniquely, the secondary carbanion in **6**-Li*_l_*·PMDTA shows both a short aryl–(α-C) distance *and* the significant retention of aromaticity.

**Figure 2:**
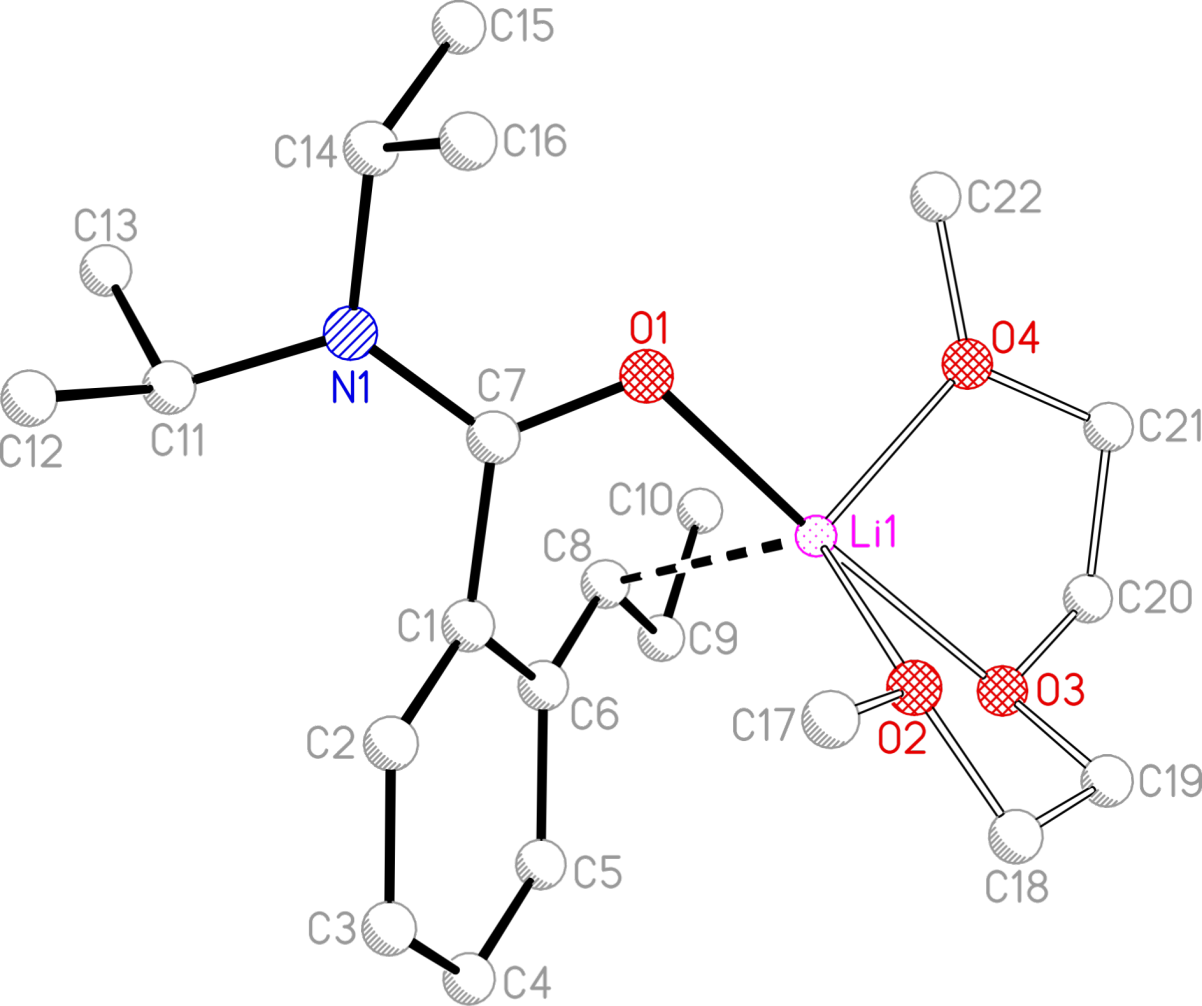
Molecular structure of **6**-Li*_l_*·DGME; H-atoms omitted. Selected bond lengths (Å) and angles (°): O1–Li1 2.001(6), O2–Li1 2.058(6), O3–Li1 2.170(6), O4–Li1 2.036(6), C8^…^Li1 2.418(6), C8–C9 1.478(5), C9–C10 1.511(6), C8–C6 1.377(5), C6–C5 1.445(5), C5–C4 1.365(5), C4–C3 1.387(6), C3–C2 1.389(5), C2–C1 1.382(5), C1–C6 1.448(5), C6–C1–C7–O1 54.2(4), C7–C1–C6–C8 12.2(5).

Similar charge delocalisation effects to those seen in **6**-Li*_l_*·PMDTA are present in **6**-Li*_l_*·DGME (Scheme 5, Figure 2 and Supporting Information File 2). Hence, the metal is wrapped by the amide and DGME oxygen atoms. Consistent with the view that oxygen is an inferior donor as compared with nitrogen (in PMDTA) and that DGME is a less sterically congesting donor additive than PMDTA, the solid-state structure of **6**-Li*_l_*·DGME allows a significantly shorter (α-C)^…^Li distance of 2.418(6) Å. As was seen, however, for tertiary carbanion systems, the close approach of these two atoms need not incur any significant reorganisation of the geometry at C8, with both C8 and C9 lying essentially in the aromatic ring plane and the C6–C8–C9 bond angle of 124.9(3)° suggesting an essentially trigonal planar sp^2^-hybridised carbanion. Akin to the short aryl–(α-C) distance in **6**-Li*_l_*·PMDTA, that in the diglyme analogue is 1.377(5) Å. However, a point of significant contrast between the structures of **6**-Li*_l_*·DGME and its PMDTA analogue is the C6–C1–C7–O1 torsional angle. Whereas this was 88.4(2)° in the latter complex and >80° in each of the previously reported secondary carbanion systems **4**-Li*_l_*·3THF [24] and **3**-Li*_l_*·PMDTA [23], the (α-C)^…^Li interaction in **6**-Li*_l_*·DGME reduces the torsional angle to only 54.2(4)°. In spite of this, at 1.485(5) Å the arene-directing group interaction is still long – arguing against any azaenolate contribution to anion stability.

### NMR spectroscopic studies

NMR spectroscopic investigation of **6**-Li*_l_*·PMDTA in [D_8_]toluene solution reveals that dissolution affords multiple solution species. Limited reformation of the starting material **6**-H is observed (species #3, see Experimental section). This phenomenon has been seen in previous solution studies of the lateral metallates of aromatic tertiary amides [23] and is assumed to reflect the sensitivity of **6**-Li*_l_*·PMDTA to traces of moisture in the deuterated solvent. However, in contrast to previous work, two forms of lateral metallate (#1 and #2, see Experimental section) are clearly seen. Based on previous work on the **4**-Li*_l_* system [64], we consider these two species to represent the *cis* and *trans* isomers of **6**-Li*_l_* that arise from slow rotation about the partially double arene–(α-C) bond in the PMDTA-solvated lithiate. The isomers exist in temperature independent major and minor forms, with the *trans* isomer assumed to dominate on steric grounds and the ratio between species approximating to 10:1:1 (#1:#2:#3). This can be clearly seen in the signals associated with the lateral alkyl chain, with a triplet and double doublet seen for the deprotonated methylene at δ 3.10 (#1) and 3.22 (#2) ppm, respectively, with **6**-H revealing a concomitant multiplet at δ 2.71–2.60 ppm. Consistent with the observation of essentially trigonal planar geometry at C8 in the solid-state structure of **6**-Li*_l_*·PMDTA (Figure 1), both solution isomers reveal significantly deshielded deprotonated carbon centres. Hence, observed shifts of δ 67.1 (#1) and 71.9 (#2) ppm contrast with that of δ 35.3 ppm for the corresponding tetrahedral α-C in **6**-H. Although depletion of aromaticity was noted in the recently reported solid-state structure of tertiary carbanion **5**-Li*_l_*·PMDTA, the character of the aromatic system in solution was less clear cut. While high-field aromatic signals (in the range of δ 6.94–5.78 ppm) were seen, computational studies suggested the close approach of the carbanionic centre and the metal, implying a more localised anionic charge in solution [20]. In the present case, the solid-state structure of **6**-Li*_l_*·PMDTA reveals the clear retention of aromatic character and, in this context, it is noteworthy that ^1^H NMR spectroscopic resonances of δ 6.90–5.58 (in major isomer #1) and 6.73–5.77 (in minor isomer #2) ppm compare closely with those seen for **5**-Li*_l_*·PMDTA.

Just as ^1^H NMR spectroscopy yields evidence for two lithiate structures in solution – in a ratio approximating to 10:1 – ^1^H,^7^Li-HOESY [65] reveals correlations between the major Li peak (δ 0.51 ppm) and ^1^H signals which, at δ 3.10 (strong correlation), 2.08 (moderate) and 6.36 ppm (weak), represent the benzylic hydrogen atom, the central Me group of PMDTA and the *ortho* hydrogen atom, respectively (see Supporting Information File 3). An analysis of the corresponding through-space displacements in the solid-state structure of **6**-Li*_l_*·PMDTA reveals H(benzylic)^…^Li and H(*ortho*)^…^Li (3.894(18) and 3.974(17) Å, respectively, viz. C8, C2) in the range expected to permit the observation of nOes [66]. This and theoretical results ([20] and see below) serve to reinforce the view that the dominant solution form of **6**-Li*_l_*·PMDTA closely resembles the structure observed in the solid state insofar as the disposition of the partial double arene–(α-C) bond is *trans*. While the solid-state structure of **6**-Li*_l_*·PMDTA also suggests the possibility of through-space interactions between the metal and NCH*Me* groups (viz. C15, C16), the well established dynamic behaviour of these latter groups [67] provides a rationale for the lack of associated nOes here. Similarly, the central PMDTA Me group (viz. C21) resides such that the associated hydrogen atoms are located 3–4 Å from the metal to which they reveal a HOESY correlation. However, while the terminal Me groups (viz. C17, C18, C24, C25) of the Lewis base also lie <4 Å from the metal, the lack of an associated correlation is consistent with greater dynamic activity. Lastly, the proposed model is consistent with the observation that the minor Li signal (δ 0.15 ppm) correlates only with the central PMDTA Me resonance at δ 2.08 ppm. *Cis*-**6**-Li*_l_*·PMDTA might be expected to reveal a correlation between Li^+^ and the β-hydrogen atoms of the deprotonated propyl group. However, both the low population of this species and the significant dynamic activity exhibited by the β-hydrogen atoms in solution – as evidenced by ^1^H NMR spcectroscopy – significantly diminish the likelihood of observing such a correlation (see Supporting Information File 3).

The study of **6**-Li*_l_*·DGME in [D_8_]toluene by NMR spectroscopy reveals data comparable to that seen for the PMDTA system. In spite of the deployment of a Na mirror to dry the deuterated solvent, significant reformation of **6**-H is observed (species #3, see Experimental section) suggesting that the **6**-Li*_l_*·DGME is highly solvent- and temperature-sensitive. The presence of hydrolyzed **6**-H is clear from the observation of the aliphatic region of the spectrum. Thus, the signals associated with the lateral alkyl chain in **6**-H reveal a multiplet (δ 2.69–2.58 ppm) for the α-CH_2_, two multiplets (δ 1.79, 1.62 ppm) for the β-CH_2_ and a triplet (δ 0.97 ppm) for Me. However, COSY reveals a laterally deprotonated species (#1) that expresses signals at δ 3.05 (α-CH), 2.35 (β-CH_2_) and 1.36 ppm (Me). Comparable with what was seen for **6**-Li*_l_*·PMDTA, the essentially trigonal planar geometry at C8 in the solid-state structure of **6**-Li*_l_*·DGME appears to be reflected in solution, with a ^13^C NMR shift of δ 64.6 ppm (α-CH, #1, cf. δ 67.1 ppm for the major isomer of **6**-Li*_l_*·PMDTA) contrasting with that of δ 35.3 ppm (α-CH_2_, #3). Lastly, the co-existence of two temperature-independent isomers of lateral lithiate (major #1, minor #2) can still be seen for the DGME system (see Experimental section). However, owing to the complexity of the spectra, the minor metallated form only expresses clear signals in the aromatic region of the ^1^H NMR spectrum. An analysis of this reveals that the ratio between the three species approximates to 1:0.15:3 (#1:#2:#3). Hence, COSY correlations reveal signals at δ 7.13–7.00 ppm for **6**-H, at δ 6.88 (1H), 6.46 (1H), 6.38 (1H), 5.63 (1H) ppm for the dominant lithiate, and at δ 6.74 (0.15H), 6.63 (0.3H), 5.69 (0.15H) ppm for the minor lithiate.

### Computational studies

**Figure 3:**
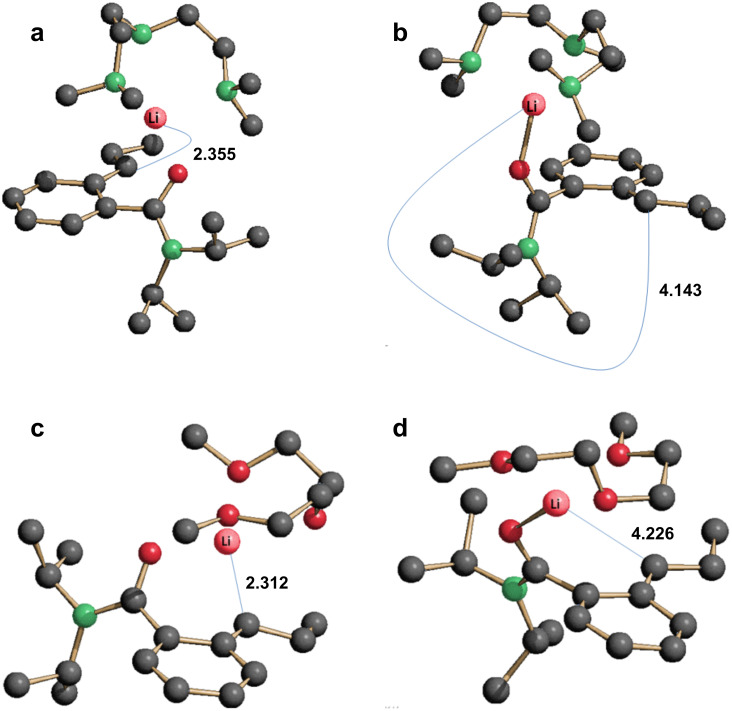
Computed minimum energy conformers (B3LYP density functional/6-311++G(2d,2p) basis set; H-atoms omitted for clarity) of *trans*-**6**-Li*_l_*·PMDTA with and without a Li–(α-C) interaction (a and b) and *trans*-**6**-Li*_l_*·DGME with and without a Li–(α-C) interaction (c and d).

The structures of **6**-Li*_l_*·L (L = PMDTA, DGME) were optimised using density functional theory (DFT) calculations. For the use of either Lewis base, two isomeric forms of **6**-Li*_l_* were modelled. These were the *cis* and *trans* isomers expected to arise from slow rotation about the partially double arene–(α-C) bond (see above). For each Lewis base, calculation of the *trans* isomer identified two minimum energy structures with very different Li–C(carbanion) distances. In the case of **6**-Li*_l_*·PMDTA these distances were 2.355 and 4.143 Å (Figure 3a and Figure 3b, respectively). The structure having the longer distance is found to be more stable by 2.6 kcal·mol^−1^, this distance being close to the experimental value (4.107(4) Å). It is also possible to compare the measured C–C bond lengths in the aromatic ring and the exogenous C–C lengths with those predicted for the two structures by using the mean unsigned error (MUE) between the calculated and experimentally observed values. We found that the MUEs are 0.010 and 0.017 Å for the PMDTA-complexed structures with the long and short metal–carbon distances, respectively. Thus, in terms of the structure of the unsaturated ring, calculation of *trans*-**6**-Li*_l_*·PMDTA favours the type of structure observed experimentally in the solid state.

A similar computational analysis to that undertaken for *trans*-**6**-Li*_l_*·PMDTA also located two structures for *trans*-**6**-Li*_l_*·DGME. However, in this case, the structure having the shorter Li–C(carbanion) distance (2.312 Å, Figure 3c) was computed to be more stable (by 2.4 kcal·mol^−1^) than that expressing a longer distance (4.226 Å, Figure 3d). This theoretical result is in line with the corresponding distance measured in the solid-state structure (2.418(6) Å). Likewise, a comparison between the measured and predicted C–C distances involving the aromatic ring gave a similar result with MUE values of 0.009 and 0.010 Å found for the structures having short and long Li–C lengths, respectively.

Having computed the most stable *trans* forms of both the PMDTA and DGME complexes of **6**-Li*_l_*, the corresponding *cis* isomers were modelled. These were found to be higher in energy; consistent with the view (see above) that they represent the less populous species in solution. Hence, *cis*-**6**-Li*_l_*·PMDTA and *cis*-**6**-Li*_l_*·DGME (see Supporting Information File 3) were 5.4 kcal·mol^−1^ and 2.2 kcal·mol^−1^ higher in energy than the minimum energy structures computed for each of *trans*-**6**-Li*_l_*·PMDTA and *trans*-**6**-Li*_l_*·DGME, respectively.

## Conclusion

*N*,*N*-Diisopropyl-2-propylbenzamide **6**-H has undergone selective directed lateral metallation in the presence of both PMDTA and DGME (= L) to give the benzyllithium **6**-Li*_l_*·L. Unlike previous examples of secondary carbanion formation under the influence of an aromatic tertiary amide directing group, the solid-state structure of **6**-Li*_l_*·PMDTA is sufficiently well resolved to reveal (i) the sp^2^ geometry of the deprotonated α-C centre, including the location of the remaining hydrogen atom and, (ii) the lengths of the arene–(α-C) bond and those that form the aromatic system. An analysis of the C–C bond lengths shows that, in contrast to all previously reported benzylic lithiates, the secondary carbanion in **6**-Li*_l_*·PMDTA shows both a short arene–(α-C) distance as well as a flat, essentially unperturbed aromatic ring. Similar observations pertain for **6**-Li*_l_*·DGME. Whereas these data contrast with those reported recently for structurally authenticated tertiary carbanions, significant similarities are seen insofar as the connectivity of the alkali metal is concerned when changing tridentate Lewis bases. Thus, whereas **6**-Li*_l_*·PMDTA fails to show a Li–(α-C) interaction in the solid state, in Li*_l_*·DGME the corresponding distance (2.406(6) Å) suggests an interaction with the amide-arene torsional angle reduced accordingly. DFT analysis successfully reproduces both the bonding patterns seen experimentally in the anionic component of **6**-Li*_l_*·L and also the varying propensity of the metal for involvement with the formally deportonated C-centre in the presence of different Lewis bases.

In contrast to previous work on carbanion formation in the presence of an aromatic N+O directing group, **6**-Li*_l_*·L dissolves in hydrocarbon media to reveal two structures in solution. Based on previous work and new DFT studies, we ascribe these as being *cis* (minor) and *trans* (major) isomers based on partial double bond character in the arene–(α-C) bond. ^1^H,^7^Li-HOESY on the PMDTA complex supports this view by revealing a correlation between the metal and the remaining benzylic hydrogen atom in this system. However, the observation that this correlation is substantially stronger than that between the metal and the remaining *ortho* hydrogen centre suggests that the structure of the major solution isomer of **6**-Li*_l_*·PMDTA may involve the metal approaching the benzylic position more closely than is seen in the solid-state structure. These data therefore suggest that, as was recently noted for the analogous tertiary carbanion complexes [20], the major form of **6**-Li*_l_*·PMDTA in solution shows strong similarities to the solid-state structure of **6**-Li*_l_*·DGME.

Further studies will seek to further probe the solution behaviour observed for **6**-Li*_l_*·L to better understand the relationship between the identity and structure of the tridentate Lewis base and the propensity of the metal and deprotonated α-C centre to interact. In particular, tandem theoretical and NMR spectroscopic studies will seek to elucidate whether polydentate Lewis bases may exhibit dynamic and variable hapticity with respect to the lithium ion in solution, potentially leading to the interconversion of different solution structures. Synthetic work will focus on employing the reported lateral metallates synthetically using a range of electrophiles. Of major interest will be the study of how steric encumbrance controls applications, particularly in the field of generating aromatics with chiral substituents at the 2-position.

## Experimental

### General synthetic and analytical details

Reactions and manipulations were carried out under dry N_2_ using standard double manifold and glove box techniques. Solvents were distilled off from sodium (toluene) or sodium-potassium amalgam (hexane) immediately before use. PMDTA and DGME were distilled from sodium at reduced pressure and stored over molecular sieves (4 Å) and *t-*BuLi (1.7 M in pentane) was purchased from Aldrich and used as received. NMR data were collected on a Bruker 500 (500.20 MHz for ^1^H, 125.80 MHz for ^13^C and 194.40 MHz for ^7^Li) FT NMR spectrometer. Spectra were obtained at 300 K and chemical shifts are internally referenced to the deuterated solvent and calculated relative to TMS for ^1^H and {^1^H}^13^C. For ^7^Li a LiCl (1 M in D_2_O) reference was employed. Chemical shifts are expressed in δ ppm. [D_8_]toluene was stored under N_2_ over a Na mirror. The following abbreviations are used for NMR spectra: s = singlet, d = doublet, t = triplet, sept = septet, (v) br = (very) broad and m = multiplet.

#### General crystallographic details

Single crystal data were collected using the ‘oil drop technique’ [68] to mount crystals on a Nonius Kappa-CCD equipped with an Oxford Cryostream low-temperature device. Structures were solved using direct methods [29], with refinement, based on *F*^2^, by full-matrix least squares [30]. Crystallographic data (excluding structure factors) have been deposited with the Cambridge Crystallographic Data Centre as supplementary publications CCDC-853884 and -853885. Copies of the data can be obtained free of charge on application to CCDC, 12 Union Road, Cambridge CB2 1EZ, UK (fax: +44 1223 336033; email: deposit@ccdc.cam.ac.uk).

**6**-H: *N*,*N*-Diisopropyl-2-propylbenzamide was prepared from *N*,*N*-diisopropylbenzamide using a modified literature method [25]. Yield 1.11 g (90%); ^1^H NMR (500 MHz, [D_8_]toluene, 300 K) δ 7.13–7.00 (m, 4H, Ar), 3.54 (sept, ^3^*J*(H,H) = 7 Hz, 1H, NCH), 2.99 (sept, ^3^*J*(H,H) = 7 Hz, 1H, NCH), 2.73–2.61 (m, 2H, ArC*H*_2_), 1.92–1.80 (m, 1H, ArCH_2_C*H*_2_), 1.70–1.58 (m, 1H, ArCH_2_C*H*_2_), 1.66 (d, ^3^*J*(H,H) = 7 Hz, 3H, NCMe), 1.64 (d, ^3^*J*(H,H) = 7 Hz, 3H, NCMe), 1.00 (d, ^3^*J*(H,H) = 7 Hz, 3H, ArCH_2_CH_2_*Me*), 0.68 (d, ^3^*J*(H,H) = 7 Hz, 6H, NCMe_2_) ppm; {^1^H}^13^C NMR (500 MHz, [D_8_]toluene, 300 K) δ 169.6 (C=O), 138.9 (C-Ar), 138.7 (C-Ar), 129.5 (CH-Ar), 127.9 (CH-Ar), 125.7 (CH-Ar), 125.0 (CH-Ar), 50.1 (NCH), 45.4 (NCH), 35.4 (Ar*C*H_2_), 24.7 (ArCH_2_*C*H_2_), 20.4 (NCMe), 20.2 (NCMe), 20.0 (NCMe_2_), 14.4 (ArCH_2_CH_2_*Me*) ppm.

**6**-Li_/_·PMDTA: *t-*BuLi (0.15 mL, 1.7 M in pentane, 0.25 mmol) was added to a solution of **6**-H (0.07 g, 0.25 mmol) in toluene/hexane (0.5:0.1 mL) containing freshly distilled *N*,*N*,*N′*,*N″*,*N″*-pentamethyldiethylenetriamine (PMDTA, 0.05 mL, 0.25 mmol) under nitrogen at −78 °C. The purple solution which resulted was warmed to room temperature before being stored at −30 °C for 3 days to yield red crystals of **6**-Li*_l_*·PMDTA. Yield 60 mg (56%); mp 101–103 °C; ^1^H NMR (500 MHz, [D_8_]toluene, 273 K, #1 **6**-Li*_l_*·PMDTA major isomer, #2 **6**-Li*_l_*·PMDTA minor isomer, #3 **6**-H) δ 7.13–7.00 (m, 0.4H, Ar#3), 6.90 (ddd, *J*(H,H) = 8, 7, 1 Hz, 1H, Ar#1), 6.73 (dd, *J*(H,H) = 8, 1 Hz, 0.09H, Ar#2), 6.69 (ddd, *J*(H,H) = 9, 6, 2 Hz, 0.09H, Ar#2), 6.65 (dd, J(H,H) = 9, 1 Hz, 0.09H, Ar#2), 6.38 (dd, *J*(H,H) = 8, 2 Hz, 1H, Ar#1), 6.36 (d, *J*(H,H) = 8 Hz, 1H, Ar#1), 5.77 (ddd, *J*(H,H) = 8, 6, 1 Hz, 0.09H, Ar#2), 5.58 (dd, *J*(H,H) = 7, 7 Hz, 1H, Ar#1), 4.78 (sept, ^3^*J*(H,H) = 6 Hz, 0.09H, NCH#2), 4.3–3.7 (v br m, 2H, NCH#1), 3.55 (sept, ^3^*J*(H,H) = 7 Hz, 0.1H, NCH#3), 3.22 (dd, ^3^*J*(H,H) = 6, 4 Hz, 0.09H, ArC*H*#2), 3.10 (t, ^3^*J*(H,H) = 6 Hz, 1H, ArC*H*#1), 3.06 (m, 0.2H, NCH#2 + NCH#3), 2.71–2.60 (m, 0.2H, ArC*H*_2_#3), 2.41 (m, 2H, ArCHC*H*_2_#1), 2.25–1.60 (v br m, 9.52H, CH_2_-PMDTA), 2.08 (s, 17.85H, Me-PMDTA), 2.02 (v br m, ~0.22H, ArCHC*H*_2_#2 or ArCHCH_2_*Me*#2), 1.92–1.80 (m, 0.1H, ArCH_2_C*H*_2_#3), 1.70–1.58 (m, 0.1H, ArCH_2_C*H*_2_#3), 1.63 (d, ^3^*J*(H,H) = 5 Hz, 0.3H, NCMe#3), 1.62 (d, ^3^*J*(H,H) = 4 Hz, 0.3H, NCMe#3), 1.60 (d, ^3^*J*(H,H) = 7 Hz, 0.27H, NCMe#2), 1.47 (v br m, ≈0.22H, ArCHC*H*_2_#2 or ArCHCH_2_*Me*#2), 1.45 (d, ^3^*J*(H,H) = 7 Hz, 0.27H, NCMe#2), 1.44 (t, ^3^*J*(H,H) = 6 Hz, 3H, ArCHCH_2_*Me*#1), 1.32–1.10 (br m, 12H, NCMe#1), 1.06 (d, ^3^*J*(H,H) = 6 Hz, 0.27H, NCMe#2), 0.98 (t, ^3^*J*(H,H) = 7 Hz, 0.3H, ArCH_2_CH_2_*Me*#3), 0.84 (d, ^3^*J*(H,H) = 7 Hz, 0.27H, NCMe#2), 0.73 (d, ^3^*J*(H,H) = 7 Hz, 0.3H, NCMe#3), 0.72 (d, ^3^*J*(H,H) = 7 Hz, 0.3H, NCMe#3) ppm; {^1^H}^13^C NMR (125 MHz, [D_8_]toluene, 273 K, #1 **6**-Li*_l_*·PMDTA major isomer, #2 **6**-Li*_l_*·PMDTA minor isomer, #3 **6**-H) δ 179.4 (C=O#1), 177.4 (C=O#2), 169.5 (C=O#3), 146.6 (C-Ar#2), 144.7 (C-Ar#1), 138.9 (C-Ar#3), 138.7 (C-Ar#3), 130.1 (CH-Ar#2), 130.0 (CH-Ar#1), 129.4 (CH-Ar#3), 127.8 (CH-Ar#3), 127.0 (CH-Ar#1), 126.7 (CH-Ar#2), 125.6 (CH-Ar#3), 124.9 (CH-Ar#3), 123.1 (CH-Ar#2), 113.9 (C-Ar#1), 112.0 (CH-Ar#1), 108.8 (C-Ar#2), 99.9 (CH-Ar#2), 96.3 (CH-Ar#1), 71.9 (Ar*C*H#2), 67.1 (Ar*C*H#1), 57.1 (CH_2_-PMDTA), 53.8 (CH_2_-PMDTA), 51.2 (NCH#1), 50.0 (NCH#3), 45.3 (NCH#3), 45.0 (Me-PMDTA), 43.8 (Me-PMDTA), 35.3 (Ar*C*H_2_#3), 26.3 (ArCH*C*H_2_#2), 24.5 (ArCH2*C*H_2_#3), 22.6 (ArCH*C*H_2_#1), 22.5 (NC*Me*#2), 21.2 (NC*Me*#1), 21.0 (NC*Me*#1), 20.4 (NC*Me*#3), 20.2 (NC*Me*#3), 20.0 (NC*Me*#3), 17.4 (ArCHCH_2_*Me*#2), 16.8 (ArCHCH_2_*Me*#1), 14.2 (ArCH_2_CH_2_*Me*#3) ppm; ^7^Li NMR (194 MHz, [D_8_]toluene, 273 K, #1 **6**-Li*_l_*·PMDTA major isomer, #2 **6**-Li*_l_*·PMDTA minor isomer) δ 0.51 (s, #1), 0.15 (s, #2) ppm; anal. calcd for C_25_H_47_LiN_4_O: C, 70.38; H, 11.10; N, 13.13; found: C, 70.34; H, 10.83; N, 12.33; crystal data for **6**-Li·PMDTA: C_25_H_47_LiN_4_O, *M* = 426.61, monoclinic, space group *P*2_1_/*n*, *a* = 9.6194(2), *b* = 29.1721(8), *c* = 10.0296(3) Å, β = 102.643(2)°, *V* = 2746.24(13) Å^3^, *Z* = 4, ρ_calcd_ = 1.032 g cm^−3^; Mo Kα radiation, λ = 0.71070 Å, μ = 0.063 mm^−1^, *T* = 180 K; 14239 data (4461 unique, *R*_int_ = 0.0477, θ < 24.40°) were collected on a Nonius Kappa CCD diffractometer. Structure solved by direct methods and refined by full-matrix least-squares on *F*^2^ values of all data giving *wR*2 = {Σ[*w*(*F*_o_^2^-*F*_c_^2^)^2^]/Σ[*w*(*F*_o_^2^)^2^]}^1/2^ = 0.1261, conventional *R* = 0. 0493 for *F* values of 2591 reflections with *F*_o_^2^ > 2σ(*F*_o_^2^), *GoF* = 0.976 for 310 parameters. Residual electron density extrema 0.159 and −0.141 eÅ^−3^.

**6**-Li*_l_*·DGME: *t-*BuLi (0.15 mL, 1.7 M in pentane, 0.25 mmol) was added to a solution of **6**-H (0.07 g, 0.25 mmol) in toluene/hexane (0.3:0.1 mL) containing diglyme (DGME, 0.04 mL, 0.25 mmol) under nitrogen at −78 °C. The resulting purple solution was warmed to room temperature and stored at −30 °C for 3 days to yield red crystals of **6**-Li*_l_*·DGME. Yield 44 mg (45%); mp <25 °C; ^1^H NMR (500 MHz, [D_8_]toluene, 253 K, #1 **6**-Li*_l_*·DGME major isomer, #2 **6**-Li*_l_*·DGME minor isomer, #3 **6**-H) δ 7.13–7.00 (m, 12H, Ar#3), 6.88 (dd, ^3^*J*(H,H) = 7, 8 Hz, 1H, Ar#1), 6.74 (d, ^3^*J*(H,H) = 8 Hz, 0.15H, Ar#2), 6.63 (m, 0.3H, Ar#2), 6.46 (d, ^3^*J*(H,H) = 7 Hz, 1H, Ar#1), 6.38 (d, ^3^*J*(H,H) = 9 Hz, 1H, Ar#1), 5.69 (m, 0.15H, Ar#2), 5.63 (dd, ^3^*J*(H,H) = 6, 7 Hz, 1H, Ar#1), 4.4–3.6 (v br m, 2H, NCH#1), 3.54 (m, 3H, NCH#3), 3.39 (t, ^3^*J*(H,H) = 4 Hz, 16H, CH_2_-DGME), 3.27 (t, ^3^*J*(H,H) = 5 Hz, 16H, CH_2_-DGME), 3.15 (s, 24H, Me-DGME), 3.08 (m, 3H, NCH#3), 3.05 (m, 1H, ArC*H*#1), 2.69–2.58 (m, 6H, ArC*H*_2_#3), 2.35 (m, 2H, ArCHC*H*_2_#1), 1.79 (m, 3H, ArCH_2_C*H*_2_#3), 1.62 (m, 3H, ArCH_2_C*H*_2_#3), 1.61 (d, ^3^*J*(H,H) = 7 Hz, 9H, NCMe#3), 1.60 (d, ^3^*J*(H,H) = 7 Hz, 9H, NCMe#3), 1.36 (t, ^3^*J*(H,H) = 7 Hz, 3H, ArCHCH_2_*Me*#1), 1.24 (br m, 6H, NCMe#1), 1.13 (br m, 6H, NCMe#1), 0.97 (t, ^3^*J*(H,H) = 7 Hz, 9H, ArCH_2_CH_2_*Me*#3), 0.73 (d, ^3^*J*(H,H) = 7 Hz, 18H, NCMe#3) ppm; {^1^H}^13^C NMR (125 MHz, [D8]toluene, 273 K, #1 **6**-Li*_l_*·DGME major isomer, #2 **6**-Li*_l_*·DGME minor isomer, #3 **6**-H) δ 179.5 (C=O#1), 177.2 (C=O#2), 169.6 (C=O#3), 145.9 (C-Ar#1), 138.7 (C-Ar#3), 138.6 (C-Ar#3), 137.5 (C-Ar#1), 130.8 (CH-Ar#2), 130.1 (CH-Ar#1), 129.4 (CH-Ar#3), 127.8 (CH-Ar#3), 127.1 (CH-Ar#1), 126.5 (CH-Ar#2), 125.6 (CH-Ar#3), 125.0 (CH-Ar#3), 122.6 (CH-Ar#2), 112.4 (CH-Ar#1), 99.2 (C-Ar#2), 97.6 (CH-Ar#1), 71.5 (CH_2_-DGME), 70.1 (CH_2_-DGME), 64.6 (Ar*C*H#1), 58.4 (Me-DGME), 50.1 (NCH#3), 45.4 (NCH#2), 35.3 (Ar*C*H_2_#3), 24.4 (ArCH_2_*C*H_2_#3), 22.3 (ArCH*C*H_2_#1), 20.4 (NC*Me*#3), 20.2 (NC*Me*#3), 20.0 (NC*Me*_2_#3), 16.6 (ArCHCH_2_*Me*#1), 14.2 (ArCH_2_CH_2_*Me*#3) ppm; ^7^Li NMR spectroscopy (194 MHz, [D_8_]toluene, 273 K, #1 **6**-Li*_l_*·DGME major isomer, #2 **6**-Li*_l_*·DGME minor isomer) δ 0.62 (s, #2), 0.26 (s, #1) ppm; anal. calcd for C_22_H_38_LiNO_4_: C, 68.19; H, 9.88; N, 3.61; found: C, 68.00; H, 9.62; N, 4.12; crystal data for **6**-Li*_l_*·DGME: C_22_H_38_LiNO_4_, *M* = 387.47, monoclinic, space group *P*2_1_/*n*, *a* = 7.8821(16), *b* = 18.975(4), *c* = 15.737(3) Å, β = 92.84(3)°, *V* = 2350.8(8) Å^3^, *Z* = 4, ρ_calcd_= 1.095 g cm^−3^; Mo Kα radiation, λ = 0.71073 Å, μ = 0.073 mm^−1^, *T* = 173 K. 13765 data (4238 unique, *R*_int_ = 0.0425, θ < 25.31°) were collected on a Nonius Kappa CCD diffractometer. The structure was solved by direct methods and refined by full-matrix least-squares on *F*^2^ values of all data to give *wR*2 = {Σ[*w*(*F*_o_^2^−*F*_c_^2^)^2^]/Σ[*w*(*F*_o_^2^)^2^]}^1/2^ = 0.2729, conventional *R* = 0. 0938 for *F* values of 2961 reflections with *F*_o_^2^ > 2σ(*F*_o_^2^), *GoF* = 1.040 for 253 parameters. Residual electron density extrema 0.729 and −0.616 eÅ^−3^.

#### Computational details

Geometry optimisation of *cis*/*trans*-**6**-Li*_l_*·L (L = PMDTA, DGME) was carried out using the B3LYP density functional and a 6-311++G(2d,2p) basis and Gaussian 09 (see Supporting Information File 3). To give free energy differences relevant to the solid state, thermodynamic corrections (at the B3LYP/6-311G(2d,2p) level) and condensed phase effects, using the CPCM model (at the B3LYP/6-311++G(2df,2dp) level) with a dielectric constant of 2.37, were included.

## Supporting Information

File 1 Crystallographic data for **1**-Li·PMDTA.

File 2 Crystallographic data for **1**-Li·DGME.

File 3 NMR spectroscopic data and DFT calculations for **6**-Li_/_·L (L = PMDTA, DGME).
