# Supplementary material for: On the control of secondary carbanion structure utilising ligand effects during directed metallation
Source: Beilstein J Org Chem. 2012 Jan 9;8:50–60. doi: 10.3762/bjoc.8.5 (PMC3302098; doi:10.3762/bjoc.8.5)
Supplement: File 3 — NMR spectroscopic data and DFT calculations for 6-Li/·L (L = PMDTA, DGME). [file Beilstein_J_Org_Chem-08-50-s003.pdf]

Supporting Information

for

On the control of secondary carbanion structure utilising ligand effects during directed metallation

Andrew E. H. Wheatley*[1], Jonathan Clayden[2], Ian H. Hillier [2], Alison Campbell Smith[1], Mark A. Vincent[2], Laurence J. Taylor[1] and Joanna Haywood[1]

Address: [1]Department of Chemistry, University of Cambridge, Lensfield Road, Cambridge, CB2 1EW, UK and [2]School of Chemistry, University of Manchester, Oxford Road, Manchester, M13 9PL, UK

Email: Andrew Wheatley - aehw2@cam.ac.uk

* Corresponding author

NMR spectroscopic data and DFT calculations for 6-Li/·L (L = PMDTA, DGME)

Contents

NMR spectroscopic characterisation of **6**-Li·PMDTA S2

NMR spectroscopic characterisation of **6**-Li·DGME S3

Computational details and results S4

References S16

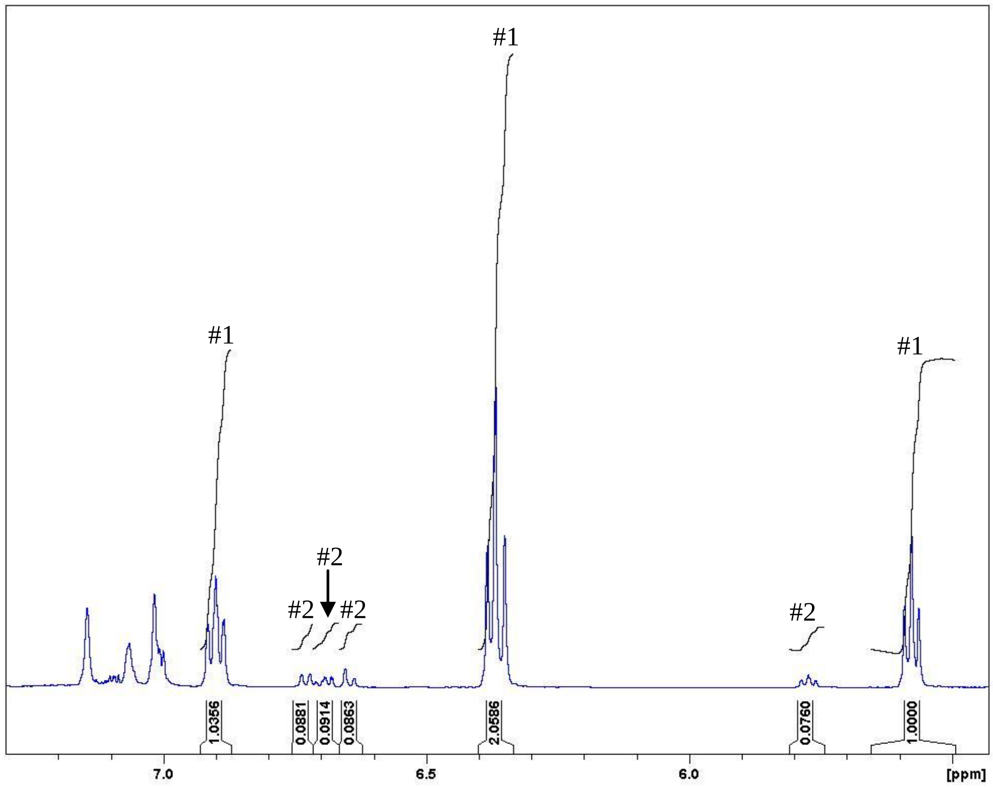

Figure S1: ^{1}H NMR spectrum of the aromatic region of **6**-Li$_{l}$·PMDTA in [D$_8$]toluene at 273 K. Unmarked signals (left) attributable to superimposed reformed **6**-H (#3) and [D$_8$]toluene.

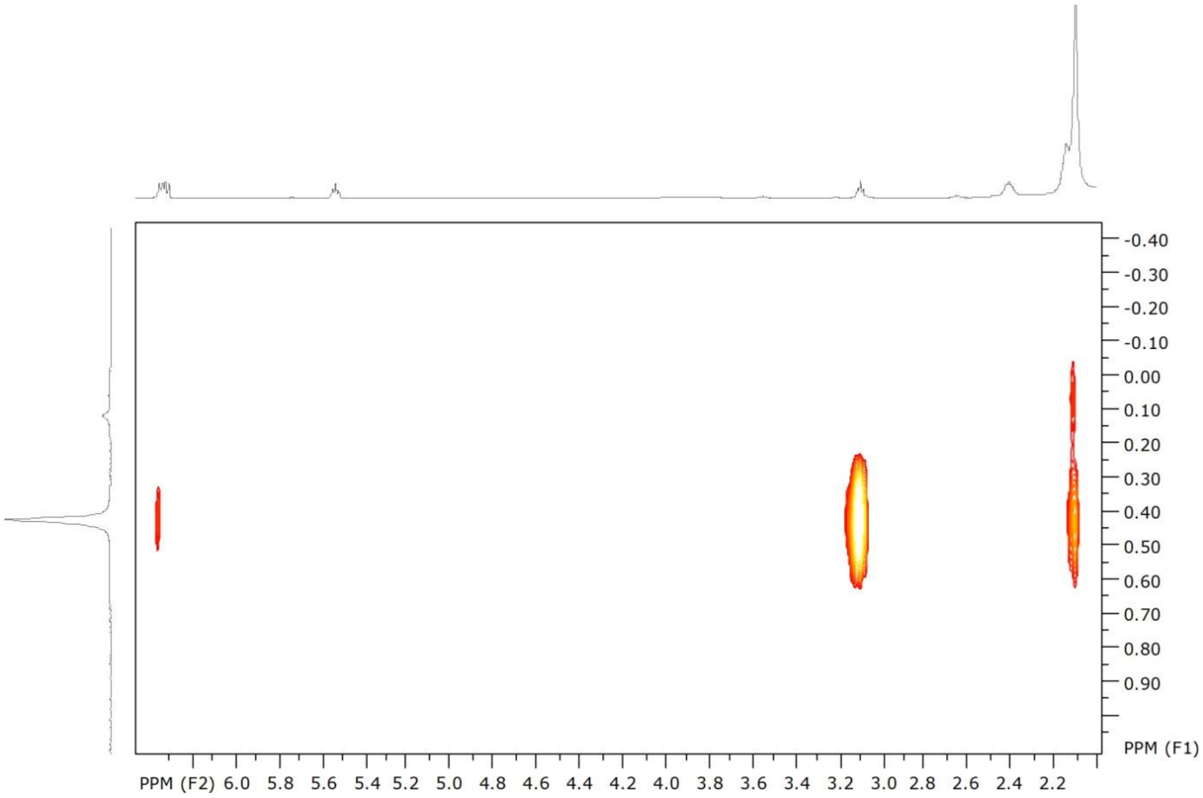

Figure S2: ^{1}H,^{7}Li-HOESY of **6**-Li$_{l}$·PMDTA in [D$_8$]toluene at 273 K (τ = 1.5 s).

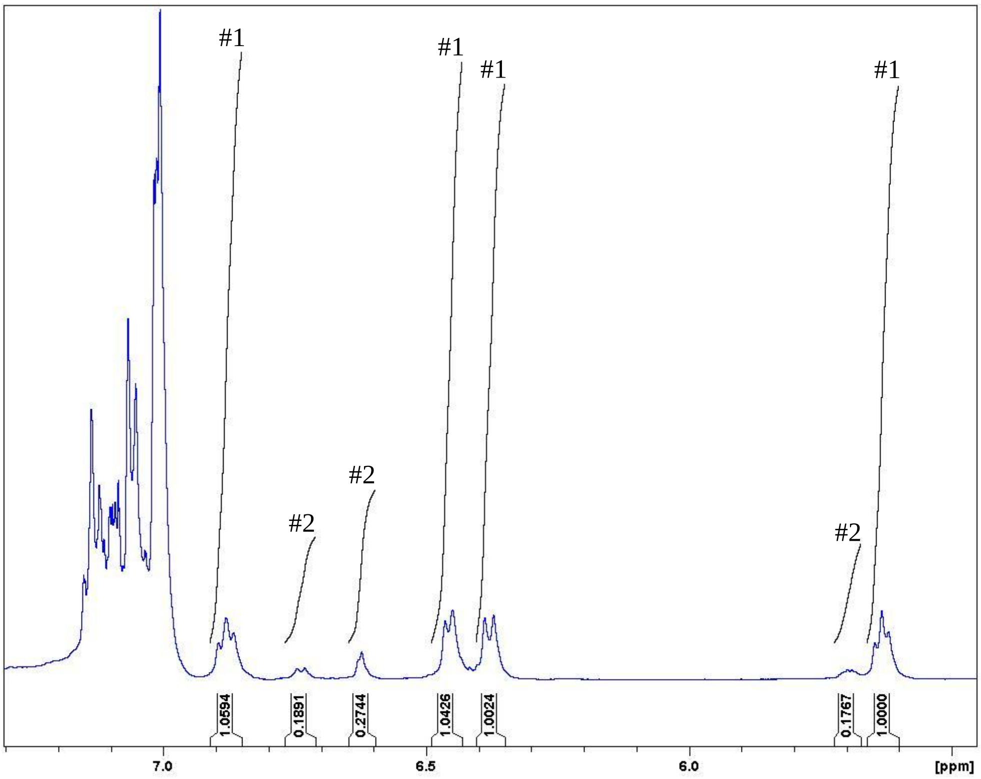

Figure S3: ^{1}H NMR spectrum of the aromatic region of **6**-Li$_l$·DGME in **[**D$_8$]toluene at 273 K. Unmarked signals (left) attributable to superimposed reformed **6**-H (#3) and **[**D$_8$]toluene.

Computational details and results

Optimisation of the geometry of *cis*/*trans*-**6**-Li_l·DGME and *cis*/*trans*-**6**-Li_l·PMDTA was carried out using the B3LYP density functional and a 6-311++G(2d,2p) basis and Gaussian 09 [1], giving the structural parameters shown below. To give free energy differences relevant to the solid state, thermodynamic corrections (at the B3LYP/6-311G(2d,2p) level) and condensed phase effects using the CPCM model (at the B3LYP/6-311++G(2df,2dp) level) with a dielectric constant of 2.37, were included.

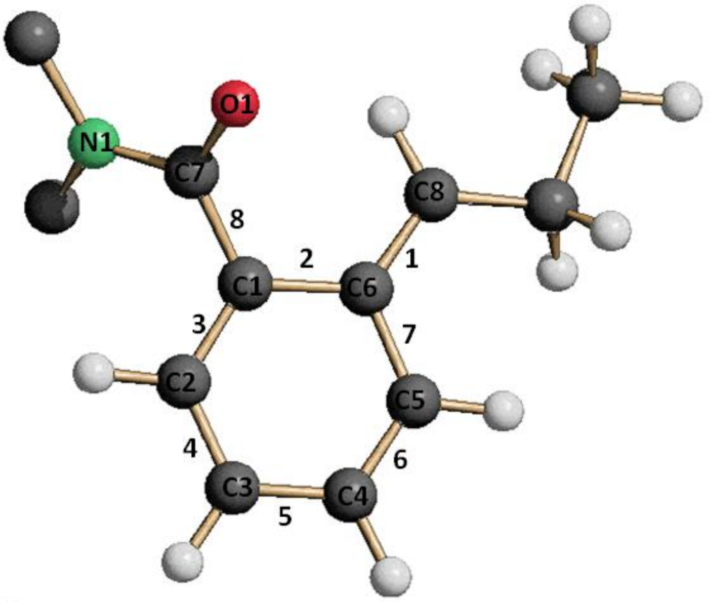

Bond	1	2	3	4	5	6	7	8
**6**-Li_l·DGME								
X-ray	1.377(5)	1.448(5)	1.382(5)	1.389(5)	1.387(6)	1.365(5)	1.445(5)	1.485(5)
DFT	1.394	1.458	1.395	1.389	1.407	1.375	1.446	1.481
**6**-Li_l·PMDTA								
X-ray	1.376(3)	1.446(3)	1.372(3)	1.405(3)	1.371(3)	1.371(3)	1.450(3)	1.493(3)
DFT	1.384	1.456	1.388	1.397	1.407	1.376	1.450	1.493

Table S1: Bond lengths (Å) of the most stable solid state forms of *trans*-**6**-Li_l·L calculated by DFT methods (–1224.81548381 au (L = DGME) and –1283.15358212 au (L = PMDTA)) compared to experiment.

Coordinates (Å) of *trans*-**6**-Li$_l$·DGME (C$^-$···Li$^+$ = 2.312 Å)

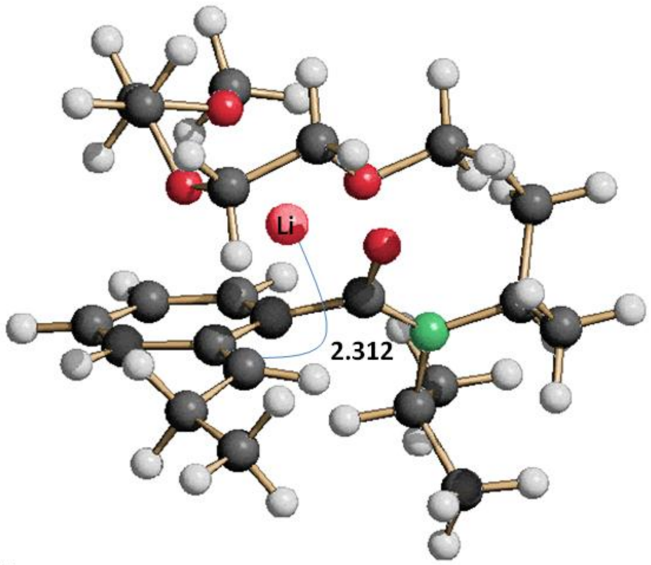

1	8	0	-0.567629	-1.019942	-0.420996
2	7	0	-2.763359	-0.582470	-0.056486
3	6	0	-1.466166	-0.177914	-0.165564
4	6	0	-1.099613	1.250984	-0.030292
5	6	0	-0.000921	1.618889	0.853947
6	6	0	0.440462	2.988222	0.708334
7	1	0	1.264836	3.328691	1.318742
8	6	0	-0.158197	3.877138	-0.152900
9	1	0	0.216480	4.893236	-0.199831
10	6	0	-1.686183	2.188760	-0.880321
11	1	0	-2.492366	1.866136	-1.528188
12	6	0	-3.150280	-1.979076	-0.381841
13	1	0	-4.232180	-1.995587	-0.263034
14	6	0	0.587661	0.751139	1.772490
15	1	0	0.090281	-0.191025	1.978496
16	6	0	-3.812398	0.283385	0.535282
17	1	0	-3.320055	1.227615	0.738664
18	6	0	-1.243403	3.502413	-0.965812
19	1	0	-1.710341	4.204700	-1.640725
20	6	0	1.559270	1.216496	2.830847
21	1	0	1.193690	2.125824	3.330310
22	1	0	2.526708	1.504530	2.397489
23	6	0	1.805967	0.155216	3.907712
24	1	0	2.533750	0.492229	4.648111
25	1	0	2.176739	-0.775717	3.471469
26	1	0	0.880897	-0.085883	4.435293
27	8	0	1.882578	0.356511	-2.033153
28	8	0	3.508965	0.125699	0.125526
29	8	0	2.223079	-2.280985	0.087810
30	6	0	0.892867	0.827505	-2.950577
31	1	0	1.326595	0.926552	-3.950174
32	1	0	0.480173	1.783779	-2.626416
33	1	0	0.102235	0.084036	-2.964518
34	6	0	3.643153	-2.301636	0.091781
35	1	0	4.010969	-2.412299	-0.934936
36	1	0	4.015129	-3.152878	0.673932
37	6	0	2.965053	1.268303	-1.872043

38	1	0	2.628830	2.150059	-1.317602
39	1	0	3.344005	1.588027	-2.848776
40	6	0	-4.319505	-0.264341	1.875579
41	1	0	-3.495331	-0.409580	2.572548
42	1	0	-4.851502	-1.210366	1.764525
43	1	0	-5.013982	0.450416	2.318368
44	6	0	-4.967273	0.551113	-0.436013
45	1	0	-4.604920	0.964071	-1.376097
46	1	0	-5.656659	1.271839	0.004603
47	1	0	-5.539049	-0.351275	-0.658270
48	6	0	-2.867692	-2.335575	-1.845586
49	1	0	-3.322146	-1.605130	-2.515103
50	1	0	-3.296081	-3.313009	-2.072575
51	1	0	-1.800140	-2.370369	-2.044543
52	6	0	4.124028	-1.007272	0.724516
53	1	0	3.829791	-0.976348	1.771723
54	1	0	5.215589	-0.945201	0.669584
55	3	0	1.280173	-0.405063	-0.105958
56	6	0	-2.582375	-3.017257	0.594618
57	1	0	-2.845432	-2.773836	1.622895
58	1	0	-1.500539	-3.069108	0.524157
59	1	0	-2.998644	-3.999703	0.364325
60	6	0	1.647200	-3.445685	-0.495122
61	1	0	2.011882	-3.586926	-1.517040
62	1	0	0.574377	-3.287802	-0.512980
63	1	0	1.887062	-4.332150	0.098521
64	6	0	4.054968	0.540146	-1.123009
65	1	0	4.383668	-0.321613	-1.709830
66	1	0	4.912996	1.200570	-0.959497

Coordinates (Å) of *trans*-**6**-Li$_l$·DGME (C^{-}···Li^{+} = 4.226 Å)

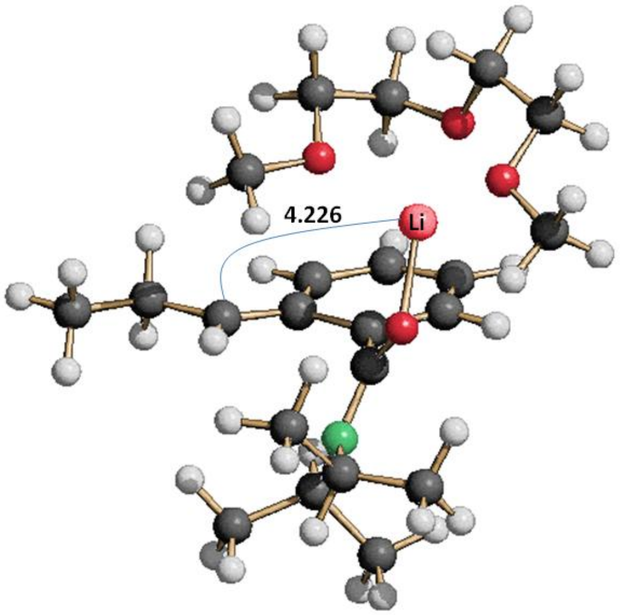

1	6	0	0.296695	-0.132585	2.147181
2	6	0	0.661766	-1.156850	3.011241
3	6	0	0.162723	-2.447398	2.733353
4	6	0	-0.608575	-2.702835	1.628049
5	6	0	-0.972371	-1.680354	0.660239
6	6	0	-0.526224	-0.334765	1.032760
7	6	0	-1.650287	-1.987847	-0.497904
8	6	0	-2.113553	-3.373830	-0.862936
9	6	0	-2.792044	-3.433475	-2.234636
10	6	0	-0.841266	0.886350	0.250603
11	8	0	0.116162	1.561359	-0.228823
12	7	0	-2.114624	1.319646	0.078139
13	6	0	-2.399918	2.547102	-0.705920
14	6	0	-1.810631	3.822733	-0.087912
15	6	0	-3.241587	0.703399	0.821257
16	6	0	-4.415175	0.332242	-0.089677
17	6	0	-3.689975	1.584980	1.992967
18	6	0	-2.031089	2.398259	-2.186614
19	3	0	1.705669	0.558553	-0.167465
20	8	0	1.791897	-0.873995	-1.559603
21	6	0	2.570422	-2.023334	-1.247416
22	6	0	3.000780	-1.908874	0.212463
23	8	0	3.322886	-0.559240	0.564444
24	6	0	4.502874	-0.013252	-0.013449
25	6	0	4.447090	1.476613	0.237868
26	8	0	3.231063	1.952215	-0.332395
27	6	0	3.015954	3.348014	-0.141993
28	6	0	1.063093	-0.987727	-2.779867
29	1	0	-0.972788	-3.708104	1.469259
30	1	0	0.387395	-3.261830	3.412934
31	1	0	0.612553	0.881709	2.369606
32	1	0	-3.481876	2.650903	-0.662651
33	1	0	-1.892754	-1.197779	-1.197000
34	1	0	-2.845063	-0.220137	1.226301
35	1	0	1.259181	-0.959921	3.889030

36	1	0	-2.816401	-3.775334	-0.117818
37	1	0	-1.276171	-4.087837	-0.861331
38	1	0	-3.116968	-4.446098	-2.479442
39	1	0	-2.114851	-3.101164	-3.025251
40	1	0	-3.671291	-2.786401	-2.266322
41	1	0	3.787992	3.928554	-0.653890
42	1	0	3.019072	3.599411	0.921954
43	1	0	2.040920	3.575306	-0.561189
44	1	0	3.423148	-2.078721	-1.934043
45	1	0	1.977816	-2.934102	-1.368982
46	1	0	4.456607	1.682029	1.313249
47	1	0	5.307790	1.971703	-0.223570
48	1	0	-4.079302	-0.277382	-0.925284
49	1	0	-4.943245	1.206500	-0.474376
50	1	0	-5.135996	-0.253667	0.480910
51	1	0	-2.856295	1.795792	2.661850
52	1	0	-4.460622	1.068321	2.566378
53	1	0	-4.112615	2.532874	1.655271
54	1	0	-2.098335	3.919649	0.958315
55	1	0	-2.192170	4.694099	-0.623482
56	1	0	-0.726357	3.821567	-0.147962
57	1	0	2.179048	-2.173792	0.873603
58	1	0	3.845562	-2.574470	0.412349
59	1	0	-2.490147	1.506383	-2.612255
60	1	0	-0.954050	2.333059	-2.316782
61	1	0	-2.394612	3.263893	-2.742720
62	1	0	1.742193	-1.185643	-3.614597
63	1	0	0.560192	-0.038055	-2.937425
64	1	0	0.316406	-1.778519	-2.697536
65	1	0	4.537545	-0.203542	-1.089237
66	1	0	5.395574	-0.444537	0.450646

Coordinates (Å) of *trans*-**6**-Li$_l$·PMDTA ($C^{-}\cdots Li^{+}$ = 2.355 Å)

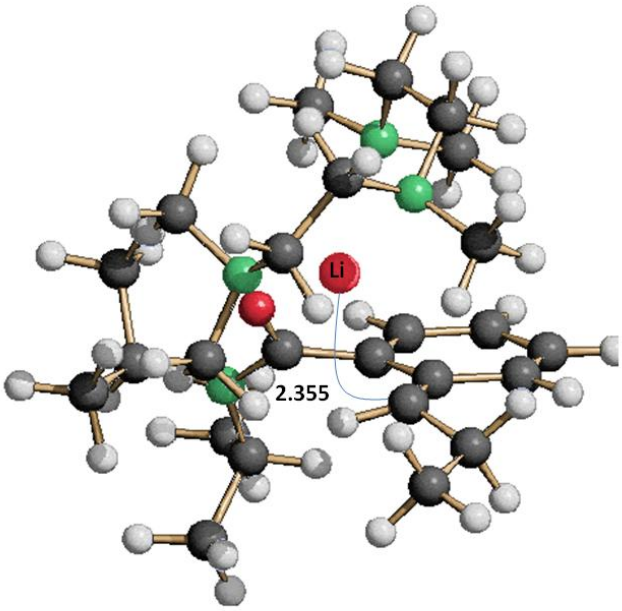

1	6	0	2.036345	3.521422	-0.800391
2	6	0	2.248359	2.152208	-0.777746
3	6	0	1.530037	1.287904	0.056150
4	6	0	0.503791	1.795258	0.952255
5	6	0	0.339608	3.228839	0.907963
6	6	0	1.066758	4.043217	0.074172
7	6	0	1.722494	-0.169491	-0.108574
8	7	0	2.983967	-0.701376	-0.098087
9	6	0	4.128660	-0.000323	0.534657
10	6	0	5.300098	0.221068	-0.428940
11	6	0	-0.267339	1.006456	1.818071
12	6	0	-1.040929	1.635113	2.958364
13	6	0	-1.577653	0.614089	3.964103
14	6	0	3.228961	-2.087846	-0.568087
15	6	0	2.611497	-3.181357	0.312989
16	8	0	0.732669	-0.914710	-0.309431
17	6	0	2.856745	-2.265193	-2.044177
18	6	0	4.587358	-0.704868	1.818650
19	3	0	-1.111555	-0.202289	-0.018811
20	7	0	-3.684484	0.178985	-0.122654
21	6	0	-3.941801	0.829017	-1.411543
22	6	0	-2.990217	0.369585	-2.512014
23	7	0	-1.575606	0.601129	-2.198024
24	6	0	-0.727808	-0.084302	-3.177912
25	6	0	-4.194588	-1.195833	-0.083649
26	6	0	-3.432240	-2.081264	0.897108
27	7	0	-2.008146	-2.226523	0.562901
28	6	0	-1.806284	-3.116274	-0.584823
29	6	0	-4.220599	0.975327	0.981070
30	6	0	-1.257350	2.036762	-2.201967
31	6	0	-1.269624	-2.752045	1.717477
32	1	0	4.308658	-2.207656	-0.502084
33	1	0	3.744720	0.972270	0.820356
34	1	0	-1.613969	-3.757784	2.000777
35	1	0	-1.395827	-2.087954	2.568875

36	1	0	-0.211251	-2.799710	1.476847
37	1	0	-1.510612	2.500202	-3.167605
38	1	0	-0.197213	2.176540	-2.012945
39	1	0	-1.796490	2.554900	-1.414046
40	1	0	1.784329	-2.165511	-2.190484
41	1	0	3.161236	-3.255981	-2.384945
42	1	0	3.363164	-1.524809	-2.663201
43	1	0	-4.128611	-1.623226	-1.082386
44	1	0	-5.262499	-1.214076	0.184317
45	1	0	2.830548	-3.013490	1.366225
46	1	0	3.028217	-4.150598	0.032497
47	1	0	1.534480	-3.218376	0.188423
48	1	0	-3.759584	1.960541	0.981051
49	1	0	-3.987246	0.507777	1.934426
50	1	0	-5.313146	1.096432	0.914627
51	1	0	-1.885261	2.235551	2.596622
52	1	0	-0.413177	2.349926	3.512855
53	1	0	3.759485	-0.814201	2.517976
54	1	0	5.362360	-0.109674	2.302973
55	1	0	5.008428	-1.691983	1.622547
56	1	0	-2.274021	-0.083962	3.494169
57	1	0	-2.103711	1.100125	4.787634
58	1	0	-0.764467	0.024265	4.392739
59	1	0	-3.491676	-1.648033	1.894564
60	1	0	-3.928085	-3.063294	0.949921
61	1	0	-3.119345	-0.698440	-2.692795
62	1	0	-3.274795	0.877731	-3.447812
63	1	0	-0.746525	-3.152976	-0.824972
64	1	0	-2.334661	-2.744260	-1.459849
65	1	0	-2.161140	-4.137260	-0.378560
66	1	0	5.782845	-0.713633	-0.718362
67	1	0	6.057030	0.839631	0.054019
68	1	0	4.978241	0.732202	-1.334556
69	1	0	-3.836105	1.902915	-1.268049
70	1	0	-4.976597	0.665992	-1.751995
71	1	0	-0.889379	0.293668	-4.198709
72	1	0	-0.937344	-1.153342	-3.170624
73	1	0	0.316293	0.059217	-2.913662
74	1	0	2.978513	1.725007	-1.453088
75	1	0	0.080361	-0.004386	2.008974
76	1	0	0.886220	5.111614	0.095629
77	1	0	2.596376	4.160922	-1.466881
78	1	0	-0.391250	3.681411	1.562274

Coordinates (Å) of *trans*-**6**-Li$_l$·PMDTA ($C^{-}\cdots Li^{+}$ = 4.143 Å)

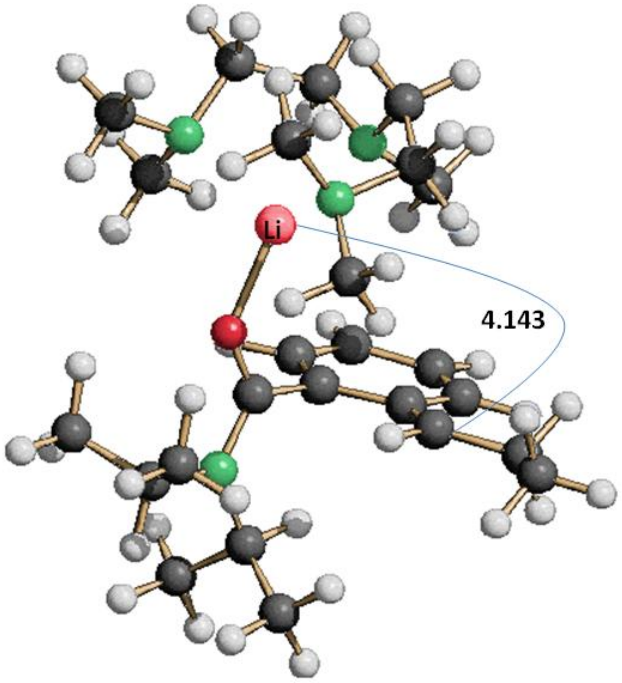

1	8	0	0.154311	-1.198500	0.318221
2	7	0	2.404545	-1.339320	0.150731
3	7	0	-2.778193	1.074129	-0.674481
4	7	0	-2.896249	-1.862505	-0.792625
5	6	0	1.226356	-0.762558	-0.176029
6	6	0	2.472386	-2.496521	1.078042
7	1	0	3.531475	-2.740274	1.127318
8	7	0	-2.220294	0.366320	2.174961
9	6	0	0.880149	0.062810	-2.470287
10	6	0	1.477742	2.687874	-1.801321
11	6	0	1.199620	0.364625	-1.153947
12	6	0	1.464010	1.727254	-0.715828
13	6	0	1.175438	2.351627	-3.100721
14	6	0	1.686549	2.083816	0.603045
15	6	0	0.838339	1.036686	-3.471448
16	6	0	3.685298	-0.859078	-0.426837
17	1	0	3.415856	0.002612	-1.028085
18	6	0	-1.066882	0.538436	3.071856
19	1	0	-1.336890	1.099653	3.977305
20	1	0	-0.273780	1.068384	2.547853
21	1	0	-0.691938	-0.438031	3.371976
22	6	0	-2.115807	-2.312448	-1.955015
23	1	0	-2.725455	-2.921567	-2.637217
24	1	0	-1.271436	-2.904839	-1.610743
25	1	0	-1.718127	-1.463005	-2.503628
26	6	0	1.758676	-3.739163	0.532364
27	1	0	0.683241	-3.588049	0.497242
28	1	0	1.969496	-4.593331	1.177997
29	1	0	2.109866	-3.980509	-0.470588
30	6	0	-3.565480	1.581158	0.464857
31	1	0	-4.408510	0.914783	0.640468
32	1	0	-3.992562	2.566557	0.235099
33	6	0	2.030296	-2.150517	2.503755
34	1	0	2.548714	-1.264414	2.867755

35	1	0	2.268384	-2.980847	3.170589
36	1	0	0.960499	-1.968024	2.545788
37	6	0	-2.019784	2.166680	-1.310550
38	1	0	-1.400524	1.772635	-2.112393
39	1	0	-1.351464	2.628260	-0.588692
40	1	0	-2.691369	2.935371	-1.717144
41	6	0	2.060326	3.475418	1.040921
42	1	0	1.324891	4.213218	0.690615
43	1	0	3.011659	3.802566	0.591367
44	6	0	4.662008	-0.385299	0.653571
45	1	0	4.198357	0.375885	1.277911
46	1	0	5.536243	0.058667	0.176855
47	1	0	5.017037	-1.200983	1.287087
48	6	0	2.185751	3.610302	2.561258
49	1	0	1.240833	3.375851	3.057171
50	1	0	2.473855	4.621487	2.854128
51	1	0	2.939715	2.924160	2.954148
52	6	0	-2.721208	1.682440	1.731560
53	1	0	-1.854357	2.312393	1.537030
54	1	0	-3.304270	2.171302	2.525483
55	3	0	-1.563302	-0.460666	0.245202
56	6	0	-4.034814	-1.020470	-1.199174
57	1	0	-4.701618	-0.938106	-0.340607
58	1	0	-4.618036	-1.498504	-2.000195
59	6	0	-3.263710	-0.402242	2.860565
60	1	0	-2.873377	-1.380109	3.138932
61	1	0	-4.124112	-0.554141	2.211716
62	1	0	-3.609733	0.102972	3.773587
63	6	0	4.310310	-1.904607	-1.355883
64	1	0	4.605927	-2.809920	-0.822339
65	1	0	5.206552	-1.491672	-1.819610
66	1	0	3.617903	-2.181691	-2.150116
67	6	0	-3.609999	0.372148	-1.664782
68	1	0	-3.024873	0.292626	-2.579142
69	1	0	-4.511562	0.946277	-1.923944
70	6	0	-3.359644	-3.030039	-0.035903
71	1	0	-4.006391	-3.684760	-0.637098
72	1	0	-3.918736	-2.709914	0.841913
73	1	0	-2.500927	-3.611308	0.296183
74	1	0	0.693788	-0.973380	-2.733527
75	1	0	1.735422	1.308819	1.358995
76	1	0	1.205614	3.125667	-3.859336
77	1	0	0.625873	0.774523	-4.497575
78	1	0	1.740034	3.712367	-1.576422

Coordinates (Å) of *cis*-**6**-Li$_l$·PMDTA (C$^-$···Li$^+$ = 4.684 Å)

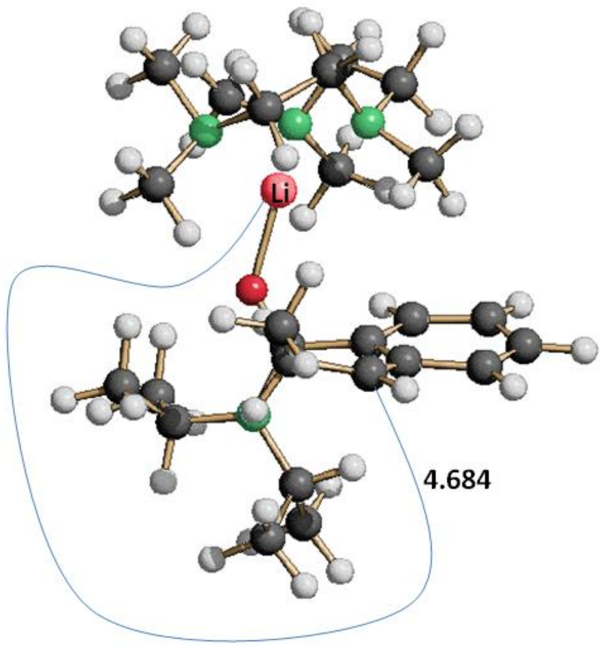

1	6	0	0.765208	3.268369	-1.497360
2	6	0	0.756771	1.887068	-1.406703
3	6	0	1.260102	1.183855	-0.301451
4	6	0	1.735960	1.900825	0.879780
5	6	0	1.825088	3.339198	0.685133
6	6	0	1.354447	3.983263	-0.422602
7	6	0	1.303665	-0.284676	-0.494930
8	7	0	2.497046	-0.927761	-0.608287
9	6	0	3.771038	-0.166729	-0.687476
10	6	0	4.293636	-0.097190	-2.127477
11	6	0	2.049872	1.365063	2.112883
12	1	0	2.419382	2.062213	2.858079
13	6	0	2.565499	-2.379272	-0.912087
14	6	0	2.038308	-3.251396	0.230911
15	8	0	0.229547	-0.937043	-0.658629
16	6	0	1.938161	-2.757151	-2.261748
17	6	0	4.839283	-0.689938	0.276734
18	3	0	-1.537293	-0.448169	-0.292094
19	7	0	-2.895991	-0.293695	-2.023196
20	6	0	-3.433779	-1.567004	-2.516398
21	6	0	-2.169437	0.373759	-3.114030
22	6	0	-3.976929	0.563629	-1.501685
23	6	0	-3.465382	1.703605	-0.621294
24	7	0	-2.658294	1.231352	0.517503
25	6	0	-1.834968	2.330881	1.057437
26	6	0	-3.485524	0.634049	1.584074
27	6	0	-2.745542	-0.476082	2.322979
28	7	0	-2.429879	-1.621208	1.446604
29	6	0	-3.622676	-2.434215	1.184403
30	6	0	-1.407624	-2.472714	2.071524
31	1	0	3.630025	-2.585903	-0.997297
32	1	0	3.522713	0.840590	-0.376259
33	1	0	-1.752372	-2.886163	3.029569

34	1	0	-0.501252	-1.899777	2.243774
35	1	0	-1.168548	-3.300584	1.406913
36	1	0	-2.826403	0.563087	-3.974511
37	1	0	-1.343159	-0.256538	-3.436178
38	1	0	-1.754779	1.318608	-2.775970
39	1	0	0.858751	-2.639976	-2.237807
40	1	0	2.175008	-3.797818	-2.491550
41	1	0	2.335749	-2.137974	-3.065030
42	1	0	-4.399972	0.235032	1.148644
43	1	0	-3.798136	1.399446	2.305578
44	1	0	2.528924	-2.999718	1.170067
45	1	0	2.237755	-4.301776	0.011706
46	1	0	0.967186	-3.122205	0.353196
47	1	0	-1.196092	2.732654	0.275912
48	1	0	-1.185665	1.965782	1.850292
49	1	0	-2.464080	3.135897	1.460948
50	1	0	4.466955	-0.713606	1.298132
51	1	0	5.699013	-0.020116	0.249153
52	1	0	5.198324	-1.685996	0.011243
53	1	0	-1.804677	-0.084760	2.708338
54	1	0	-3.337699	-0.797517	3.192514
55	1	0	-4.653277	-0.072450	-0.930644
56	1	0	-4.573242	0.990499	-2.321332
57	1	0	-3.367296	-3.249922	0.509845
58	1	0	-4.405796	-1.841810	0.717341
59	1	0	-4.030274	-2.868013	2.108752
60	1	0	4.573290	-1.079618	-2.511984
61	1	0	5.181601	0.534863	-2.165102
62	1	0	3.544558	0.335535	-2.789619
63	1	0	-2.834933	2.369125	-1.208271
64	1	0	-4.325104	2.303592	-0.289329
65	1	0	-4.131298	-1.425161	-3.353995
66	1	0	-3.958012	-2.088231	-1.718135
67	1	0	-2.615076	-2.197656	-2.859737
68	1	0	0.416823	1.306200	-2.255235
69	6	0	1.852527	-0.040050	2.592810
70	1	0	1.440216	5.062045	-0.481334
71	1	0	0.431565	3.772947	-2.392784
72	1	0	2.262987	3.917936	1.489604
73	6	0	1.438037	-0.104156	4.070577
74	1	0	1.090267	-0.537272	1.985917
75	1	0	2.745927	-0.674189	2.481491
76	1	0	1.326262	-1.134934	4.417324
77	1	0	2.189273	0.374732	4.701491
78	1	0	0.495472	0.420981	4.239209

Coordinates (Å) of *cis*-**6**-Li$_l$·DGME (C$^-$···Li$^+$ = 2.367 Å)

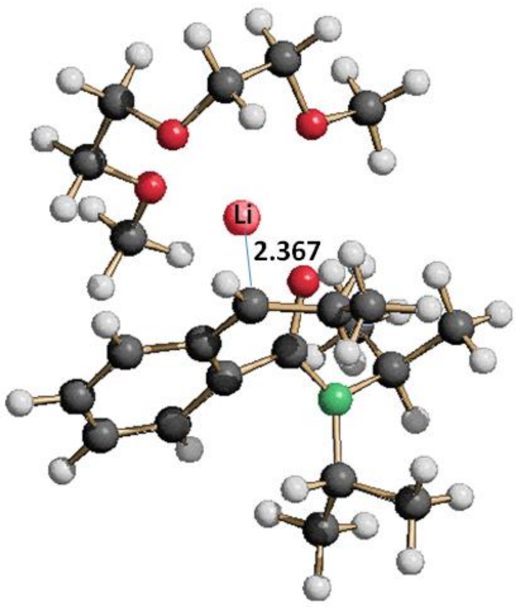

1	6	0	-1.085870	3.685001	-0.029003
2	6	0	0.098263	3.829743	0.736513
3	6	0	0.686732	2.754008	1.338040
4	6	0	0.131716	1.416838	1.279383
5	6	0	-1.014454	1.271090	0.384377
6	6	0	-1.599550	2.416374	-0.193145
7	6	0	-1.354278	-0.050364	-0.159993
8	7	0	-2.658940	-0.427835	-0.363145
9	6	0	-3.778972	0.199339	0.376110
10	6	0	-4.850700	0.783969	-0.552090
11	6	0	0.679070	0.409191	2.062494
12	1	0	1.518110	0.700950	2.685651
13	6	0	-2.980286	-1.625404	-1.175800
14	6	0	-2.553409	-2.954602	-0.536557
15	8	0	-0.427119	-0.826689	-0.525078
16	6	0	-2.487164	-1.498626	-2.622147
17	6	0	-4.402127	-0.754935	1.404359
18	3	0	1.389159	-0.280706	-0.087446
19	8	0	2.361011	-2.133244	-0.149333
20	6	0	1.838503	-3.212592	-0.917784
21	6	0	3.775534	-2.156072	-0.032226
22	6	0	4.187869	-0.958715	0.808044
23	8	0	3.595331	0.243973	0.336564
24	6	0	4.180053	0.812309	-0.831285
25	6	0	3.128265	1.681755	-1.477077
26	8	0	2.018283	0.845857	-1.789734
27	6	0	1.023976	1.517341	-2.568621
28	1	0	1.575313	2.896839	1.941250
29	1	0	0.541226	4.811620	0.855182
30	1	0	-2.444854	2.284757	-0.855240
31	1	0	-4.068090	-1.637327	-1.223687
32	6	0	0.018759	-0.891556	2.431979
33	1	0	-3.331875	1.017724	0.929464
34	1	0	-1.548259	4.536763	-0.506543
35	1	0	1.446245	1.823338	-3.530254

36	1	0	0.632786	2.387117	-2.038823
37	1	0	0.220992	0.805454	-2.729098
38	1	0	4.227834	-2.129050	-1.030265
39	1	0	4.108067	-3.077413	0.459808
40	1	0	2.811906	2.478821	-0.797183
41	1	0	3.534318	2.133349	-2.388868
42	1	0	-3.646288	-1.137009	2.089478
43	1	0	-4.902066	-1.602024	0.932851
44	1	0	-5.149951	-0.219611	1.990879
45	1	0	-4.417309	1.464249	-1.283320
46	1	0	-5.580568	1.341268	0.035917
47	1	0	-5.395197	0.008117	-1.092853
48	1	0	-2.839934	-0.569506	-3.070135
49	1	0	-2.875106	-2.327937	-3.215886
50	1	0	-1.401590	-1.517950	-2.671293
51	1	0	3.817767	-1.078666	1.824801
52	1	0	5.279489	-0.880915	0.841312
53	1	0	-2.943822	-3.050716	0.475103
54	1	0	-1.472073	-3.033125	-0.490137
55	1	0	-2.944016	-3.785323	-1.127859
56	1	0	2.269815	-3.215240	-1.923333
57	1	0	0.767053	-3.057226	-0.982057
58	1	0	2.050266	-4.168854	-0.431855
59	1	0	4.490393	0.033952	-1.533170
60	1	0	5.057000	1.411019	-0.563326
61	6	0	0.372629	-1.339198	3.855345
62	1	0	-1.070440	-0.782288	2.354326
63	1	0	0.253946	-1.718234	1.745740
64	1	0	-0.103354	-2.289585	4.105939
65	1	0	1.451541	-1.472119	3.966483
66	1	0	0.058431	-0.596796	4.590518

References

1. Gaussian 09, Revision A.1, Frisch, M. J.; Trucks, G. W.; Schlegel, H. B.; Scuseria, G. E.; Robb, M. A.; Cheeseman, J. R.; Scalmani, G.; Barone, V.; Mennucci, B.; Petersson, G. A.; Nakatsuji, H.; Caricato, M.; Li, X.; Hratchian, H. P.; Izmaylov, A. F.; Bloino, J.; Zheng, G.; Sonnenberg, J. L.; Hada, M.; Ehara, M.; Toyota, K.; Fukuda, R.; Hasegawa, J.; Ishida, M.; Nakajima, T.; Honda, Y.; Kitao, O.; Nakai, H.; Vreven, T.; Montgomery, Jr., J. A.; Peralta, J. E.; Ogliaro, F.; Bearpark, M.; Heyd, J. J.; Brothers, E.; Kudin, K. N.; Staroverov, V. N.; Kobayashi, R.; Normand, J.; Raghavachari, K.; Rendell, A.; Burant, J. C.; Iyengar, S. S.; Tomasi, J.; Cossi, M.; Rega, N.; Millam, N. J.; Klene, M.; Knox, J. E.; Cross, J. B.; Bakken, V.; Adamo, C.; Jaramillo, J.; Gomperts, R.; Stratmann, R. E.; Yazyev, O.; Austin, A. J.; Cammi, R.; Pomelli, C.; Ochterski, J. W.; Martin, R. L.; Morokuma, K.; Zakrzewski, V. G.; Voth, G. A.; Salvador, P.; Dannenberg, J. J.; Dapprich, S.; Daniels, A. D.; Farkas, Ö.; Foresman, J. B.; Ortiz, J. V.; Cioslowski, J.; Fox, D. J. Gaussian, Inc., Wallingford CT, 2009.
